# Harmful Algal Bloom–Associated Illnesses in Humans and Dogs Identified Through a Pilot Surveillance System — New York, 2015

**DOI:** 10.15585/mmwr.mm6643a5

**Published:** 2017-11-03

**Authors:** Mary Figgatt, James Hyde, David Dziewulski, Eric Wiegert, Scott Kishbaugh, Grant Zelin, Lloyd Wilson

**Affiliations:** ^1^New York State Department of Health; ^2^CDC/CSTE Applied Epidemiology Fellowship Program; ^3^New York State Department of Environmental Conservation.

Cyanobacteria, also known as blue-green algae, are photosynthetic, aquatic organisms found in fresh, brackish, and marine water around the world (*1*). Rapid proliferation and accumulation of potentially toxin-producing cyanobacteria characterize one type of harmful algal bloom (HAB). HABs have the potential to cause illness in humans and animals (*2*,*3*); however, the epidemiology of these illnesses has not been well characterized. Statewide in 2015, a total of 139 HABs were identified in New York, 97 (70%) of which were confirmed through laboratory analysis; 77 independent beach closures were ordered at 37 beaches on 20 different bodies of water. To better characterize HAB-associated illnesses, during June–September 2015, the New York State Department of Health (NYSDOH) implemented a pilot surveillance system in 16 New York counties. Activities included the collection of data from environmental HAB reports, illness reports, poison control centers, and syndromic surveillance, and increased outreach to the public, health care providers, and veterinarians. During June–September, 51 HAB-associated illnesses were reported, including 35 that met the CDC case definitions*; 32 of the cases occurred in humans and three in dogs. In previous years, New York never had more than 10 HAB-associated illnesses reported statewide. The pilot surveillance results from 16 counties during a 4-month period suggest that HAB-associated illnesses might be more common than previously reported. 

Exposure to HABs through contact, inhalation, or ingestion of contaminated water can cause illness in humans and animals (*2*,*3*). Signs and symptoms associated with HAB exposure have occurred from exposure to cyanobacteria in situations in which toxins were not detected (*4*,*5*). Symptoms associated with HAB exposure can include skin reactions, eye irritation, ear irritation, liver damage, and gastrointestinal, respiratory, and neurologic signs and symptoms (*1*–*3*).

“One Health” is a concept that encompasses the interrelatedness of human and animal health and the environment and recognizes that health outcomes might be optimized through multidisciplinary collaboration. The application of a One Health approach to HABs might result in the development of improved public health prevention and response efforts (*6*,*7*). To better understand the occurrence of HABs and the epidemiology of cyanobacteria HAB-associated illnesses NYSDOH implemented a pilot surveillance system in 2015, applying a One Health approach in a subset of counties overseen by local health departments (LHDs).

NYSDOH selected 16 (26%) of 62 New York counties for participation in the pilot surveillance system based on the likelihood of an HAB occurrence within their jurisdiction and the LHDs’ interest in participation. Public health workers from each participating county attended a training webinar and received an electronic package of surveillance tools, including health outreach documents for the general public, health care providers, and veterinarians; human and animal illness questionnaires; and illness reporting forms adapted from CDC’s One Health Harmful Algal Bloom System materials (*8*). During June–September 2015, HAB-associated human and animal illnesses and environmental HAB occurrences reported by LHDs and the New York State Department of Environmental Conservation (NYSDEC) were submitted to NYSDOH.

NYSDEC was the main source of information regarding environmental HAB occurrence, location, and laboratory confirmation. A program within NYSDEC evaluates bodies of water for HABs in response to reports of possible HABs from the public, various park staffs, and lake associations. NYSDEC notified NYSDOH of any possible or laboratory-confirmed HABs within 24 hours so that a response (e.g., closing the beach) could be implemented promptly. NYSDEC also notified the relevant LHDs and lake associations, when applicable.

When there is visual evidence of an HAB (e.g., visible scum or discolored water consistent with an HAB) at a regulated bathing beach in New York, the jurisdiction with authority over the beach requires it to be closed to swimming, wading, and other water contact. Visual evidence, as opposed to toxin monitoring, is used to trigger beach closures because of the risk for illness from both toxin-producing and nontoxin-producing HABs and the variability in the location and length of HAB occurrences. In such instances, water contact was prohibited until the HAB was no longer visible in the beach area for at least 1 day, and testing with commercially available algal toxin-detecting test strips had determined that concentrations of cyanobacterial toxins were below the recommended guidance value of 10 *μ*g/L (*9*). Using the combination of visualization and test strip approach allows for more immediate protective measures than laboratory confirmation of HABs, which might take several days to complete. Visual evidence in water bodies other than bathing beaches is also used as a trigger for LHDs to alert the public via social media, websites, press releases, and advisory signs.

As part of the pilot surveillance system, outreach materials were disseminated through NYSDOH and NYSDEC websites, a veterinary magazine, and presentations and flyers at various meetings involving lake and water quality associations. Outreach materials encouraged members of the public who had a suspected exposure to report symptoms to their LHD. Additional surveillance activities were implemented by NYSDOH when an HAB was identified that posed a substantial public health concern affecting drinking water or recreational water activities. Such surveillance activities included 1) monitoring hospital syndromic surveillance data for patients who displayed symptoms consistent with HAB exposure, 2) providing educational materials to health care providers and veterinarians near the sites of HABs, and 3) coordinating with poison control centers to notify NYSDOH if suspected HAB exposure calls were received.

HAB-associated illnesses were voluntarily reported to NYSDOH through participating counties, NYSDEC, the New York State Office of Parks, Recreation and Historic Preservation, and a veterinary office. Each report of illness was investigated using a questionnaire administered by either the LHD or NYSDOH and assessed using the CDC case definitions for HAB-associated illness. Case definitions are specific to animals and humans and take into consideration information such as environmental or visual evidence of an HAB, confirmation via toxin-detecting test strips, or laboratory confirmation (i.e., via microscopy, enzyme-linked immunosorbent assay, liquid chromatography mass spectrometry, or density of blue green chlorophyll) along with clinical evidence of HAB exposure (e.g., consistency of symptoms and onset of illness after exposure). During June–September 2015, NYSDOH received 51 HAB-associated illness reports from the pilot surveillance system, 35 (68.8%) of which met the CDC case definitions, including 32 in humans and three in dogs. Among those patients with such data available, median age of the humans was 24 years (2–63 years) and median age of the dogs was 7 years (2–12 years); 18 (56%) of the humans and one of the dogs were female. One (3%) of the 32 human cases was classified as confirmed, 20 (57%) as probable, and 11 (31%) as suspected. All three canine cases were classified as probable.

Among cases that occurred in humans, skin problems were reported by 22 (69%) patients (Table). No hospitalizations or deaths were reported among humans. All human cases were associated with exposure to HABs in recreational water. Recreational activities included swimming (28 patients; 88%); wakeboarding, jet skiing, waterskiing or tubing (seven; 22%); boating (seven; 22%); wading (six; 19%); and using personal watercraft (four; 13%). All three affected dogs had gastrointestinal symptoms, and two were hospitalized; the third dog’s symptoms resolved without intervention, and none of the dogs died. One dog was associated with human cases. The dogs were exposed to HABs in recreational water and, according to their owners, might have ingested water or algae.

**TABLE:** Illness outcomes and number of commonly reported symptoms for harmful algal bloom–associated illnesses in humans (N = 32) and dogs (N = 3),* identified through a pilot surveillance system — New York, June–September 2015

Outcome/Symptom	No. of human cases	No. of canine cases
**Illness outcome**		
Outpatient care**^†^**	17	0
Hospitalized	0	2
**Commonly reported symptoms^§^**		
Skin problems (e.g., rash)	22	0
Respiratory (e.g., cough or dry cough)	16	0
Gastrointestinal (e.g., abdominal pain, diarrhea, nausea, vomiting)	15	3
Other symptoms (e.g., chills, muscle aches, or watery eyes)	13	0
Fatigue/General weakness	11	2
Sore throat	11	0
Neuroglogic (e.g., headache or seizure)	6	2
**Exposure type/Setting^§^**		
Swimming	28	3
Boating	7	0
Wading	6	3
Personal watercraft (e.g., kayak or canoe)	4	0
Tubing/Waterskiing	2	—
Jet skiing	1	—
Drinking untreated water	0	3

* Among 32 human cases, 11 were suspected, 20 were probable, and one was confirmed. All three canine cases were probable. https://www.cdc.gov/habs/pdf/ohhabs-case-and-event-definitions-table-3-14-17.pdf.

^†^ Includes reported visit to an emergency department, urgent care clinic, or primary care.

^§^ Categories are not mutually exclusive. Patients could have multiple symptoms and exposures.

## Discussion

HAB-associated illness reports made to NYSDOH before 2015 never exceeded 10 statewide in any given year, whereas 51 illness reports were made through a pilot surveillance system in 16 New York counties during June–September 2015. Of the 51 reports identified through the pilot surveillance system, 35 were considered cases of HAB-associated illness that met the CDC case definition, suggesting that such illnesses might be underreported.

The findings in this report are subject to at least three limitations. First, self-reported illnesses and exposures can be subject to inaccuracy and bias. Second, surveillance for HAB-associated illnesses is difficult because HAB–associated signs and symptoms are nonspecific (e.g., skin rash, nausea, or diarrhea); the frequent lack of data showing these illnesses are causally linked to HAB exposure leads to a case definition with low specificity. Finally, all of the pilot counties had experienced at least one HAB in a water body in the previous season. Therefore, the pilot counties might represent communities more aware of HABs and their potential health effects.

Developing partnerships with local, state, and federal partners using a One Health approach assisted NYSDOH in successfully implementing a pilot surveillance system through identification of environmental HAB events, illness identification and reporting, and outreach to the public, lake associations, physicians, and veterinarians. NYSDEC’s HAB monitoring program supported NYSDOH efforts to implement an HAB-associated illness surveillance system through the identification of HABs and public outreach. Collaboration with other organizations, such as lake associations, medical and veterinary organizations, and poison control centers, might have helped to establish reporting of HAB-associated illnesses. Tools are available online to guide the implementation of HAB-associated illness surveillance or to develop prevention and response efforts for other state and local health departments (*8*).

**Summary**

**What is already known about this topic?**

Recreational water exposure to excessive growths of cyanobacteria, called harmful algal blooms (HABs), can result in adverse health effects in humans and animals. Although HAB-associated illnesses and outbreaks have been documented in recent years, the extent and severity of these illnesses have not been well described.

**What is added by this report?**

In 2015, New York implemented a pilot cyanobacteria HAB-associated illness surveillance system in 16 counties. During June–September, 51 human and canine HAB-associated illnesses were reported, including 35 that met the CDC case definition.

**What are the implications for public health practice?**

HAB-associated illnesses might be more common than has been previously reported. Establishing working relationships with local health departments, environmental agencies, medical and veterinary organizations, poison control centers, and lake associations can provide important partnerships for public health response to HABs.

## Footnotes

* Suspected human and animal cases required exposure to water, algae, seafood, or dietary harmful algal bloom (HAB) sources; signs or symptoms following an HAB exposure; and a public health assessment of whether the illness was likely HAB-related. For animals, assessments also could have been completed by qualified nonpublic health entities that were identified by state or federal agency partners. Probable human and animal cases met the suspected case definition and, in addition, were required to have a professional medical diagnosis or have been supported by either observational or HAB environmental laboratory data. Confirmed human and animal cases met the suspected case definition and were required to have HAB clinical laboratory data plus either 1) a professional medical diagnosis or rule-out of other causes of illness; or 2) a professional medical diagnosis, rule-out of other causes of illness, and HAB environmental laboratory data. https://www.cdc.gov/habs/pdf/ohhabs-case-and-event-definitions-table-3-14-17.pdf.
